# Positional transfer accuracy of titanium base implant abutment provided by two different scan body designs: an invitro study

**DOI:** 10.1186/s12903-023-03399-9

**Published:** 2023-10-11

**Authors:** Rania E. Ramadan, Mahmoud Khamis Abdel Razek, Faten S. Mohamed, Rania A. Fahmy, Mervat E. Abd-Ellah

**Affiliations:** 1 Department of Prosthodontics, Faculty of Dentistry, Alexandria University, Alexandria, Egypt; 2 Department of Oral Medicine and Periodontology, Faculty of Dentistry, Alexandria University, Alexandria, Egypt

**Keywords:** Scan body, Healing abutment-scan peg, Positional transfer accuracy, Titanium base abutment, Meteorology software

## Abstract

**Background:**

The variabilities in design and material of scan bodies have a major role in the positional transfer accuracy of implants. The purpose of this invitro study was to compare the 3D transfer accuracy (trueness and precision) of titanium base (TB) abutment position provided by 2 different scan bodies: one-piece scan body (SB) in comparison to two-piece healing abutment and scan peg (HA-SP).

**Methods:**

A maxillary model with a dummy implant in the 2nd premolar (Proactive Tapered Implant; Neoss) was 3D printed and TB (Ti Neolink Mono; Neoss) was tightened on the implant and scanned by using a laboratory scanner (inEos X5; Dentsply Sirona) (reference scan). An SB (Elos Medtech) and an HA-SP (Neoss) were subsequently connected to the implant and were scanned 10 times each by using the same scanner (test scans). All the scans were exported as STL files and imported into CAD software where the TBs were formed. Test scans were superimposed on reference scans for transfer accuracy analysis using 3D metrology software (GOM Inspect; GOM GmbH) in terms of angular deviation in vertical and horizontal directions, linear deviation in each XYZ axis of TBs and total linear deviation in all axes. Statistical analysis was done using independent sample t test. When Levene’s test for equality of variances was significant, Welch’s t-test was used. (*P* value < 0.05)

**Results:**

Significant differences were found amongst the tested groups in both angular and linear deviation in terms of trueness with less deviation values for the SB group (*P* < 0.001). For the precision, significant differences were found amongst the tested groups in angular deviation in vertical direction with less deviation value for the SB group compared to HA-SP group *(P* < 0.001). However, no significant difference was found between the tested groups regarding the angular deviation in horizontal direction (*P* = 1.000). Moreover, significant differences were found amongst the tested groups in linear deviations with less linear deviations in XYZ axes for SB compared to HA-SP group (*P* = 0.020, < 0.001, = 0.010 respectively).

**Conclusions:**

SB showed less angular and linear deviation values in the 3D positional transfer of TB than HA-SP indicating higher degree of accuracy of SB.

## Background

Transfer accuracy of implant position is considered a crucial step in implantology as it is a fundamental requirement in the long-term effectiveness of implant-supported restorations [1–3]. Because an implant’s mobility is 10 times lower than that of a natural tooth, implant prosthodontics requires extremely high accuracy in transferring the 3D implant position intraorally to a model virtually. Inaccurate implant position’s transfer may eventually result in biological, mechanical, and functional complications [4–7].

Scan bodies are described by Mizumoto and Yilmaz [2] as “complex implant position transfer devices.“ Scan bodies may be attached to an implant analog extraorally on a stone cast after conventional impression and scanned by a laboratory scanner (indirect digital impression) or may be attached to an implant intraorally and scanned by an intraoral scanner (direct digital impression) [8–11]. The direct digital impression offers certain benefits over the indirect approach including the direct transfer of intraoral data to the laboratory, and the elimination of potential errors associated to dimensional changes of the conventional impression and stone cast [2, 12].

Scan bodies from different manufacturers come in varying designs, sizes, implant connection types and are made of different materials. They diverge in several ways. They may be made of titanium, PEEK, or a combination [2]. Also, they may be designed as one or two pieces, used single or several times, screw-retained or snap-on (friction grip), radiolucent or radiopaque, having a sandblasted or coated surface, tall or short, with a wide or narrow width, and with a simplified or complicated design [13]. These differences can play a role in the transfer accuracy of implant position [5–7].

Commonly, most scan bodies are one-piece of cylindrical or conical design with little or no taper and their geometry usually does not simulate the natural tooth’s emergence profile. All of them consist of an upper scan region, a middle body, and a base that is connected to the implant [2]. The part that is scanned by the intraoral scanner is the scan region, however the base should be adequately seated on the implant connection. Thus, the design of scan base depends on the type of implant connection and dimension of platform. In addition, the base material may have an impact on the fit when screwing on the implant, whereas the surface characteristics of the material used for the scan region may affect how many spots the intraoral scanner can detect digitally [2, 3, 14].

However, other scan bodies are 2-pieces like HA-SP (Neoss) that is formed of a contoured HA and a scannable body called SP. The SP is maintained by friction between an indentation on the SP and a vertical depression in the contoured HA, thus acting as anti-rotational feature [15–18]. HA is made entirely of PEEK, and SP is made of medical grade acrylic-based polymer. It does not need to be sprayed with nonreflective powder required for metals during scanning and contrary to titanium scan bodies, and the scan is captured at the HA’s level [15–18]. Furthermore, the soft tissues can be properly contoured for appropriate emergence profile, which makes it easier for the soft tissues to accept the definitive implant-supported restoration. The implant and peri-implant tissues are scanned together, and the CAD software includes the contoured HA, allowing its duplication on the definitive restoration. Consequently, reducing the dental chairside time and improving the satisfaction of patients and clinicians [19].

Evaluating the 3D transfer accuracy of TB abutment position is always a very important objective as it corresponds to the accuracy of implant position. However, it is challenging to conduct it in a clinical study due to the lack of a reference model since a patient’s jaw cannot be analyzed using high-precision laboratory scanners [4, 20]. In addition, in vitro studies led to a coordinate-based evaluation [21]. Therefore, the current invitro study was conducted.

Based on International Organization for Standardization 5725-1 [22], accuracy includes trueness in addition to precision. Trueness is the proximity to the actual position of a reference object, while precision is the proximity of same object’s position to one another which is attained through repetitive measurements.

**Fig. 1:**
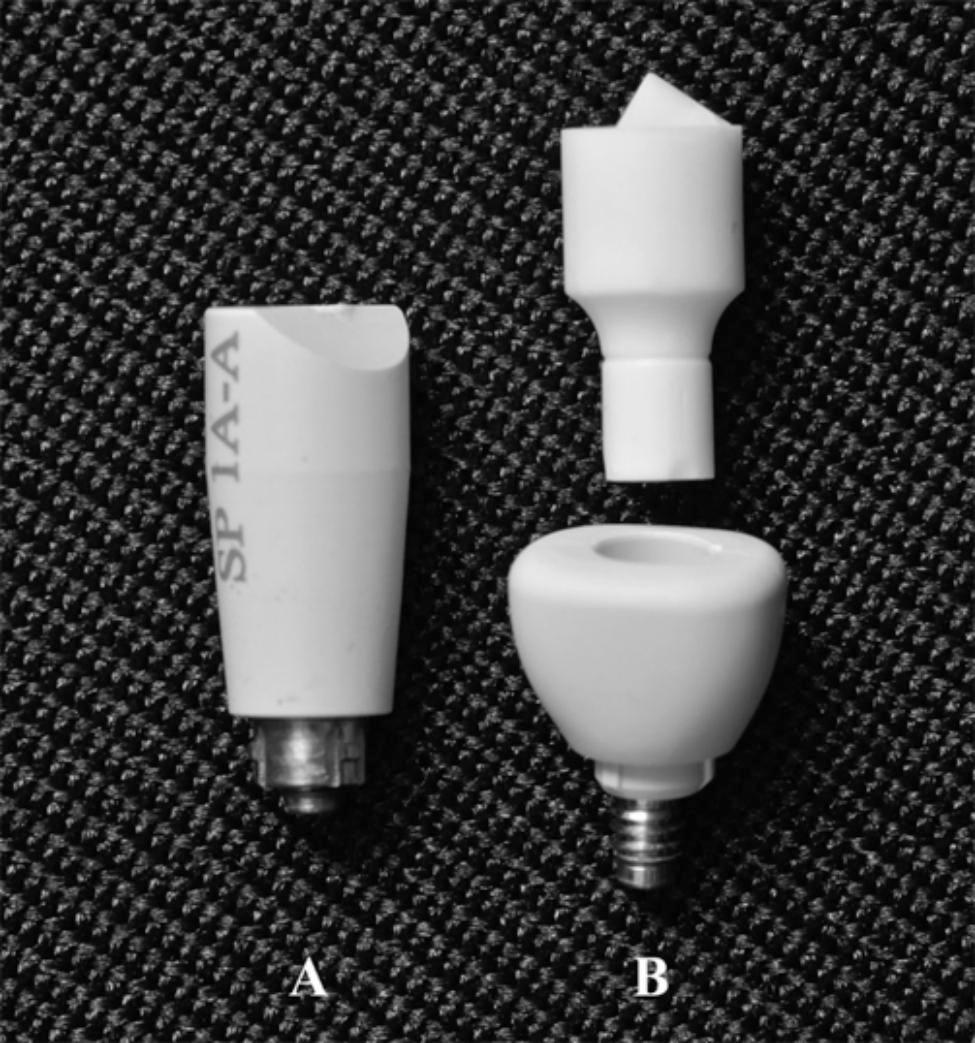
Investigated scan bodies. (**A**) One-piece SB, (**B**) Two-piece HA-SP.

The purpose of this invitro study was to compare the 3D transfer accuracy of TB abutment position provided by 2 different designs of scan bodies: one-piece SB in comparison to two-piece HA-SP. (Fig. 1) The null hypothesis was that no difference would be found in the 3D transfer accuracy (trueness and precision) of TB position using the mentioned scan bodies.

## Materials and methods

### Sample size calculation

Based on previous studies [5, 16] a sample size of 10 scans for each group was calculated with a software program (G*Power v3.1.9.2; Heinrich Heine University Düsseldorf) [23] supposing a 95% level of confidence and 80% power. Following the preliminary findings, it was determined that the sample size permitted the identification of high statistic significant differences; hence, it was decided that no additional scans were required.

### Fabrication of 3D printed implant model

**Fig. 2:**
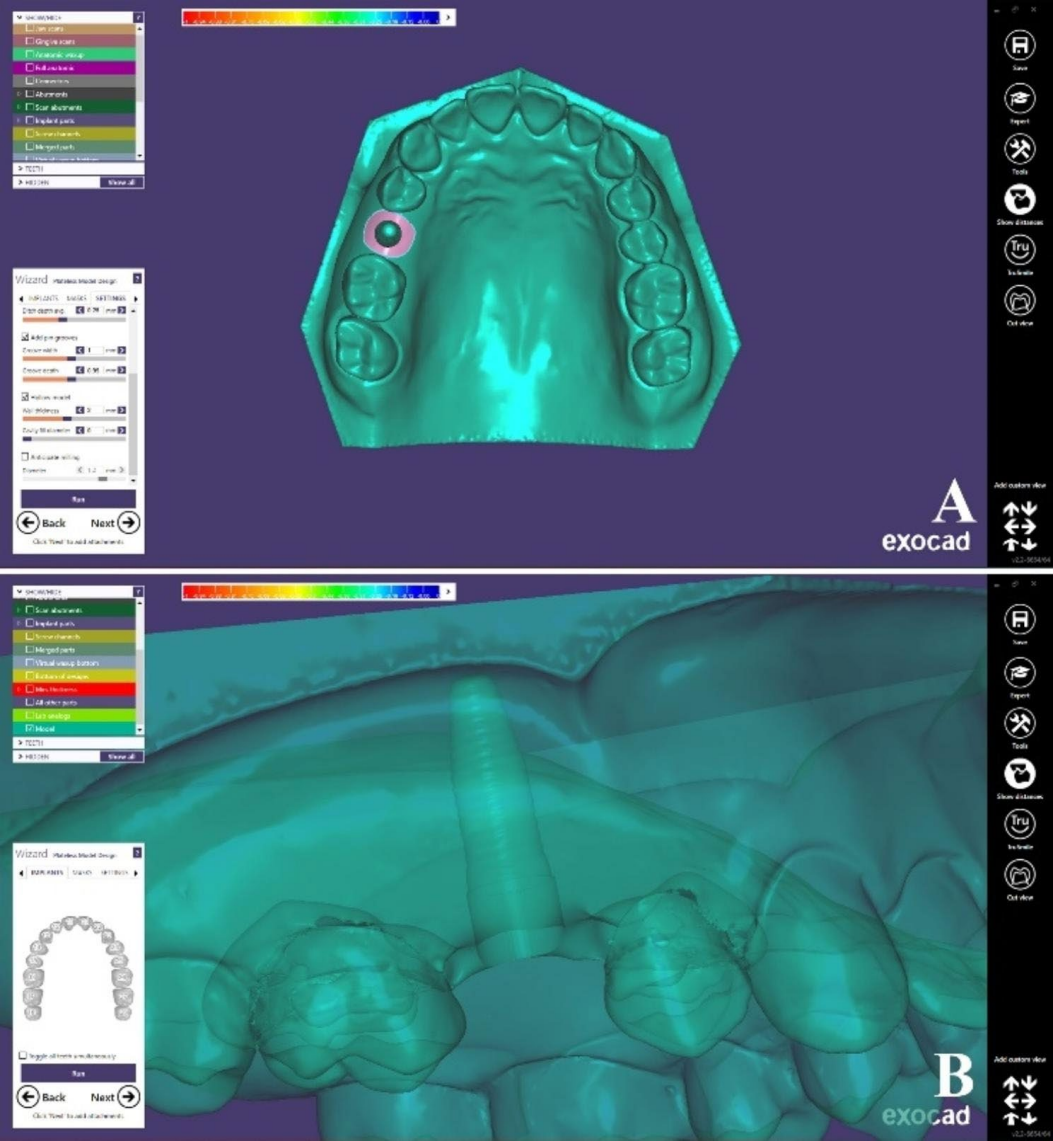
Design of maxillary model with an implant site in 2nd premolar, (**A**) Occlusal view, (**B**) Lateral view

**Fig. 3:**
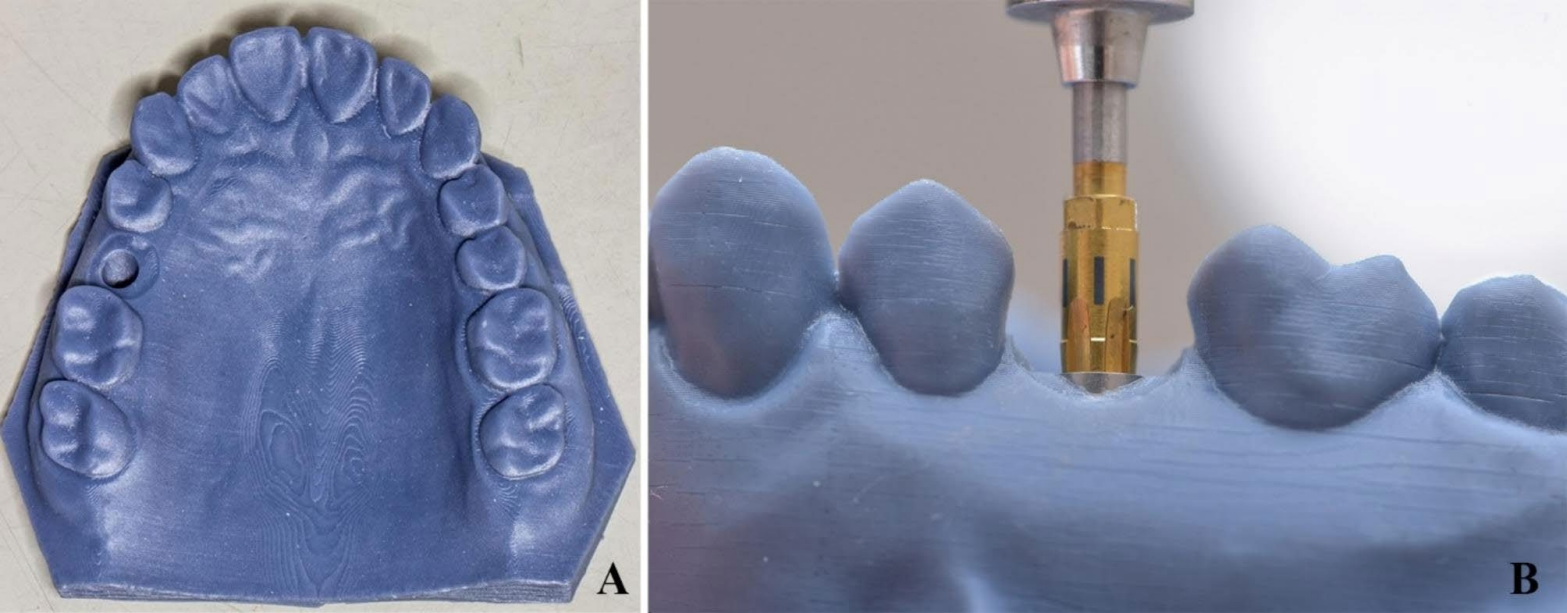
**A**. 3D printed implant model, **B.** The vertical mark on the screwdriver was positioned mid-buccally during implant placement

A maxillary resin model with an implant site at the right 2nd premolar was designed on CAD design software (Dental DB 3.0 Galway; Exocad GmbH) using the model creator module and the Neoss implant library as shown in Fig. 2. Then, the model was additively manufactured using 3D printer (Dent2 3D printer; Mogassam Co LLC). (Fig. 3A) A dummy implant (4.0 × 11 mm) (Proactive Tapered Implant; Neoss) was cemented in the corresponding site using cyanoacrylate adhesive (Super Bonder; Henkel Loctite Corp) so that the vertical mark on the screwdriver was positioned mid-buccally during implant insertion for correct further orientation of TB abutment and scan bodies [19]. (Fig. 3B)

### Data acquisition

**Fig. 4:**
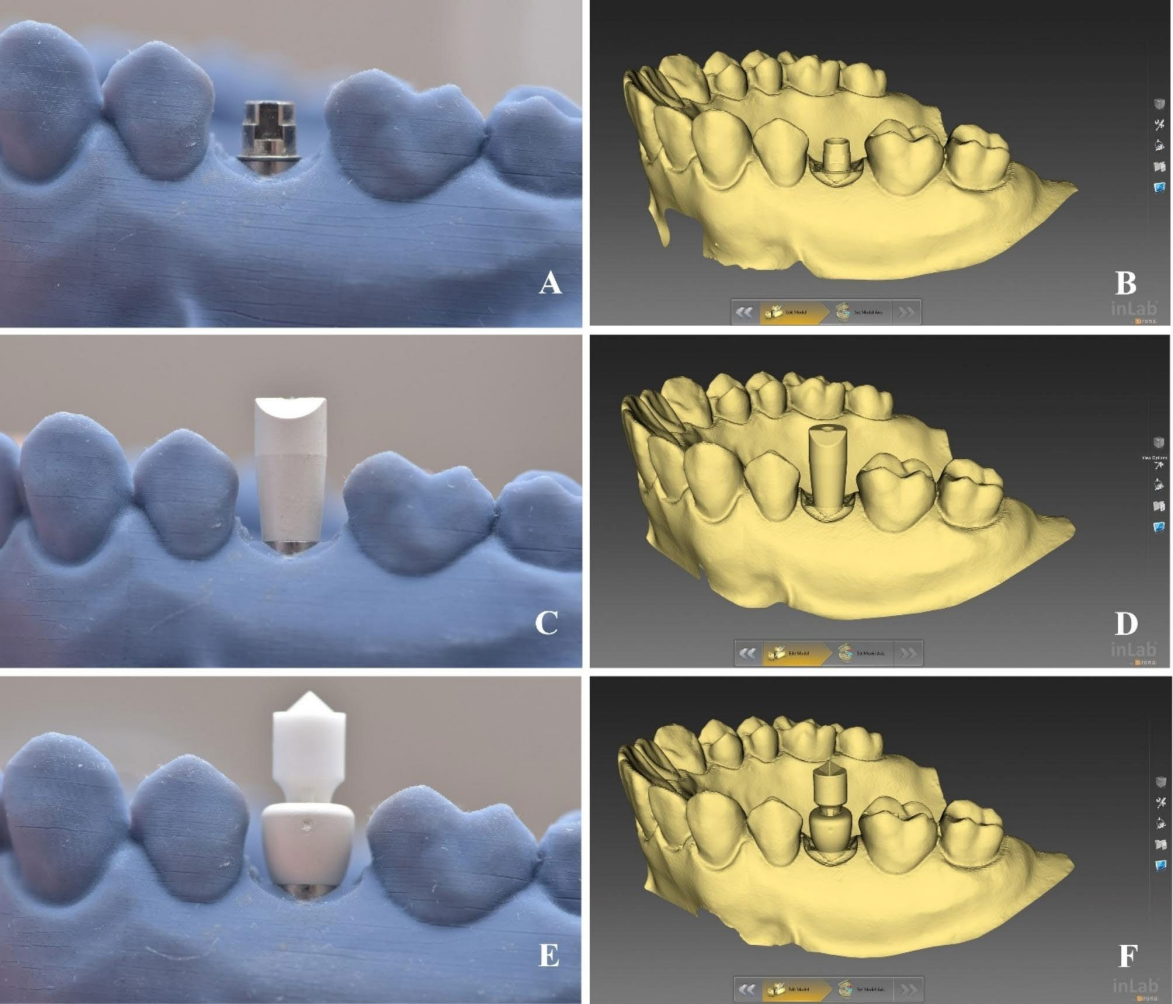
Data acqusition (**A**) TB abutment tightened on the implant so that the flat surface is positioned buccally, (**B**) Desktop scanning of TB, (**C**) SB tightened on the implant, (**D**) Desktop scanning of model with SB, (**E**) HA-SP tightened on the implant, (**F**) Desktop scanning of model with HA-SP.

A hexed TB abutment (Ti Neolink Mono; Neoss) was screwed on the implant by a manual torque wrench to 32 Ncm using the gold prosthetic screw so that the flat surface was positioned buccally. (Fig. 4A) The model with TB was scanned by using a high precision laboratory scanner (inEos X5; Dentsply Sirona) [24, 25] and was exported as an STL file (STL-1) that was further used to get the reference scan. (Fig. 4B)

Then, the one-piece SB (Elos Medtech) was tightened on the implant to 10 Ncm so that the beveled surface was placed buccally, and the model was scanned by the same scanner 10 consecutive times without taking the model out of the scanning tray in between scans (STL-2). (Fig. 4C, D)

Following that, the SB was removed, and the premolar HA (Healing Abutment-Scan Peg; Neoss) was inserted so that the vertical groove on the HA was placed buccally and tightened to 10 Ncm. Then, the SP was secured on the HA by means of a vertical indentation on the SP and a vertical groove in the HA, thus acting as an anti-rotational means and enabling proper seating of the SP in the HA. The model with HA-SP was scanned 10 consecutive times with the same scanner without taking the model out of the scanning tray in between scans (STL-3). (Fig. 4E, F)

All the scans were made on the same day of printing the model [26]. Each scan was checked for imperfections, especially on the investigated scan bodies’ surfaces. A scan was accepted when no significant flaws or voids were found [27].

### Reference scan

**Fig. 5:**
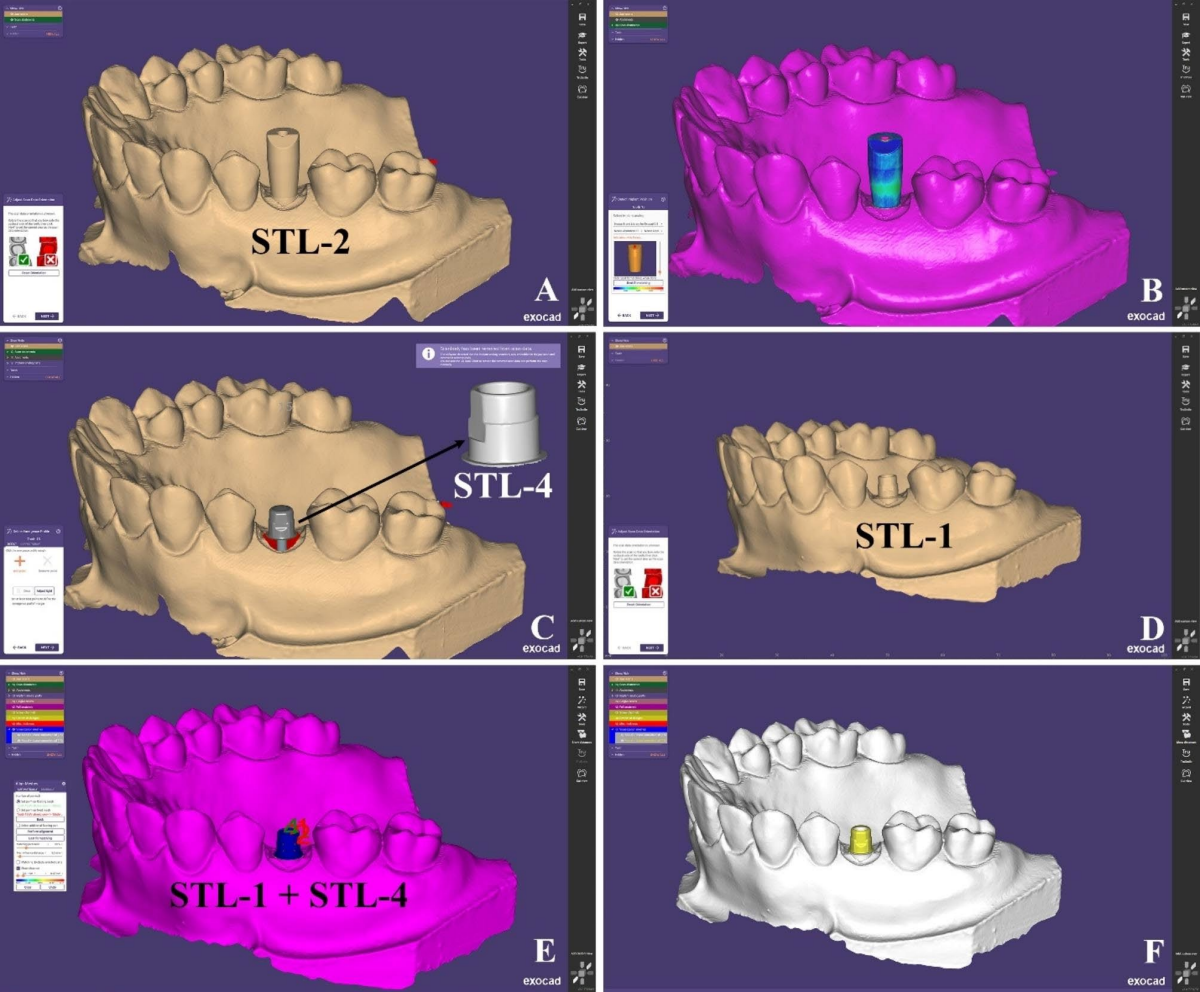
Steps to obtain the reference scan (**A**) STL-2 was imported to CAD software, (**B**) “Best-fit matching” between SB in STL-1 and SB in CAD library, (**C**) TB was formed and exported solely as STL-4, (**D**) STL-1 was imported to CAD software, (**E**) STL-1 and STL-4 were aligned together through fixing points, (**F**) Reference scan was formed with well-defined TB structure

The reference scan was obtained through the following steps:

Step 1: An STL file of the scanned model with SB (STL-2) was imported to the CAD design software. (Fig. 5A)

Step 2: The SB corresponding to hexed TB used was selected from Neoss library (Neolink mono 3.5–5.5 IO) on the CAD software and superimposed using the “best-fit matching” tool. (Fig. 5B)

Step 3: The TB was formed, trimmed from the model, and exported solely as an STL-4. (Fig. 5C)

Step 4: An STL file of scanned model with TB (STL-1) was imported to the software (Fig. 5D) together with STL-4 which was subsequently aligned through fixing points on the 2 meshes (Fig. 5E) to get a well-defined structure of hexed TB. This STL was considered as the reference STL. (Fig. 5F).

### SB test scans

**Fig. 6:**
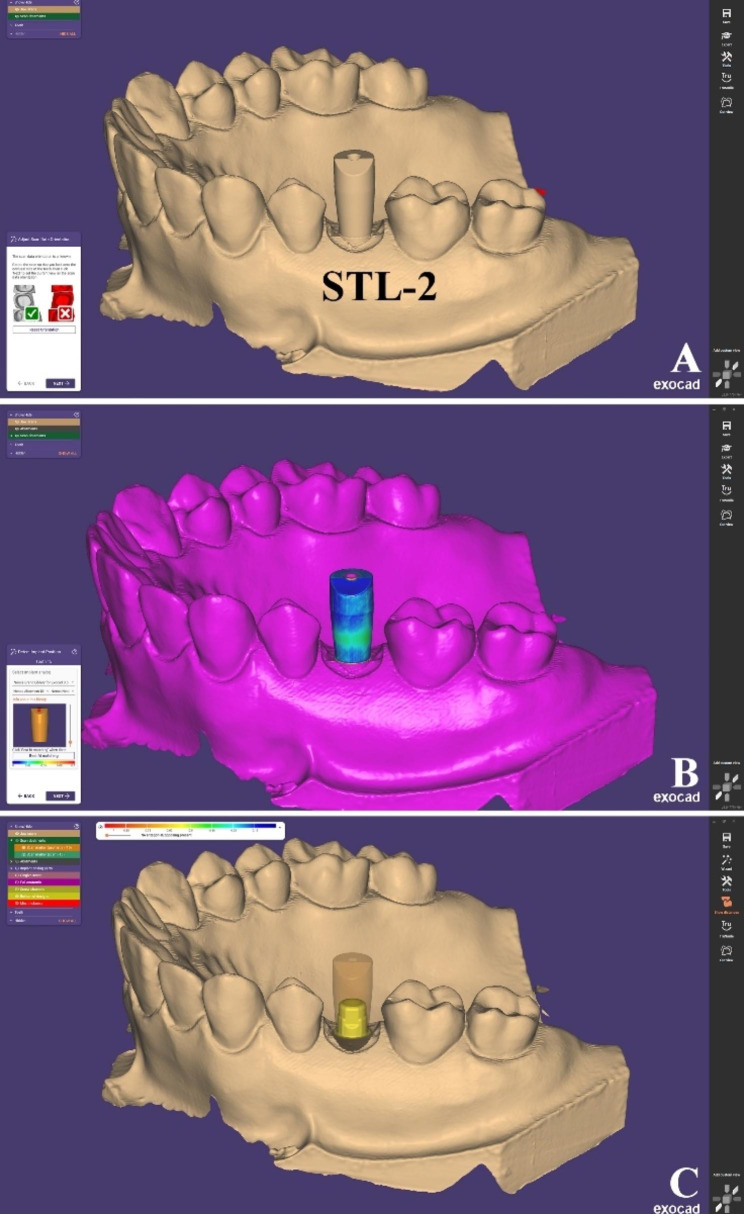
Steps to obtain SB test scans (**A**) STL-2 was imported to CAD software, (**B**) Virtual alignment of SB using the “best-fit matching” tool, (**C**) TB position corresponding to SB was formed (SB test scan)

An STL file of scanned model with SB (STL-2) was imported to the CAD software and superimposed using the “best-fit matching” tool with the SB in the library to form the virtual position of the TB (Fig. 6).

### HA-SP test scans

**Fig. 7:**
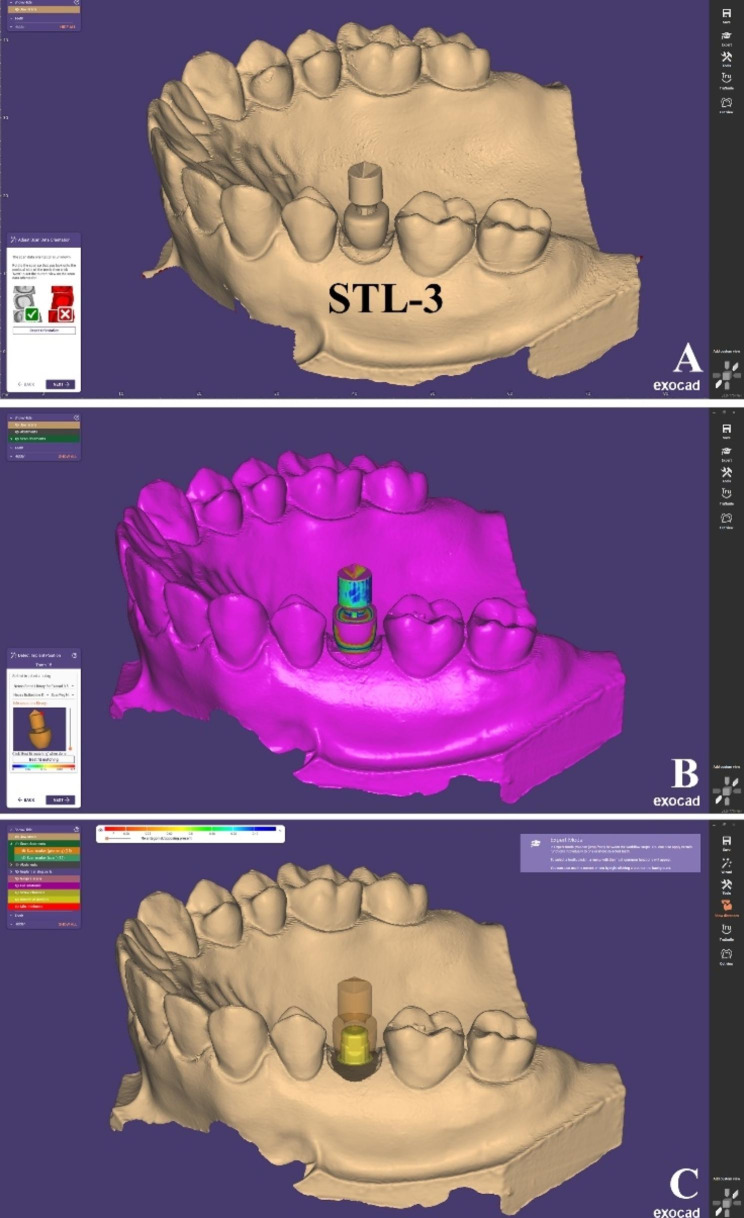
Steps to obtain HA-SP test scans (**A**) STL-3 was imported to CAD software, (**B**) Virtual alignment of HA-SP using the “best-fit matching” tool, (**C**) TB position corresponding to HA-SP was formed (HA-SP test scan)

AnSTL file of scanned model with HA-SP (STL-3) was imported to the CAD software. The scan marker used was selected from the library (Scan peg premolar Neolink) on the software and superimposed using the “best-fit matching” tool to form the virtual position of the TB. (Fig. 7)

The accuracy of CAD software “best-fit matching” for each scan was checked by the section view that could show how much the scan marker in CAD library corresponds with the used scan bodies and the color map that was displayed where the blue color means the zero deviation [2, 13]. For each test scan in each group, this alignment process was carried out once. After being aligned, the model was exported as a single STL file in the form of a model with TB in position. For further accuracy analysis, a total of 10 aligned models were produced for each group having the virtual position of the corresponding TBs.

### Accuracy analysis

Positional transfer accuracy was analyzed with 3D metrology software (GOM Inspect; GOM GmbH). The measurements were conducted by a single operator (RER). The measurements were done 3 times and the average was obtained. There was no need for intra observer reliability because every step made was based on computer-assisted algorithms with reproducible outcomes [28].

Regarding trueness analysis, angular and linear deviations between reference and test scans were measured [22]. Each test scan was compared to the reference scan as SB test scan having the position of TB formed by the SB was compared to the reference scan having the original position of TB. Similarly, HA-SP test scan having the position of TB formed by HA-SP was compared to the reference scan having the original position of TB.

For precision analysis, the variance of angular and linear deviations within each test group was measured [22]. In other words, the repeatability of SB or HA-SP to form the same position of TB every time.

**Fig. 8:**
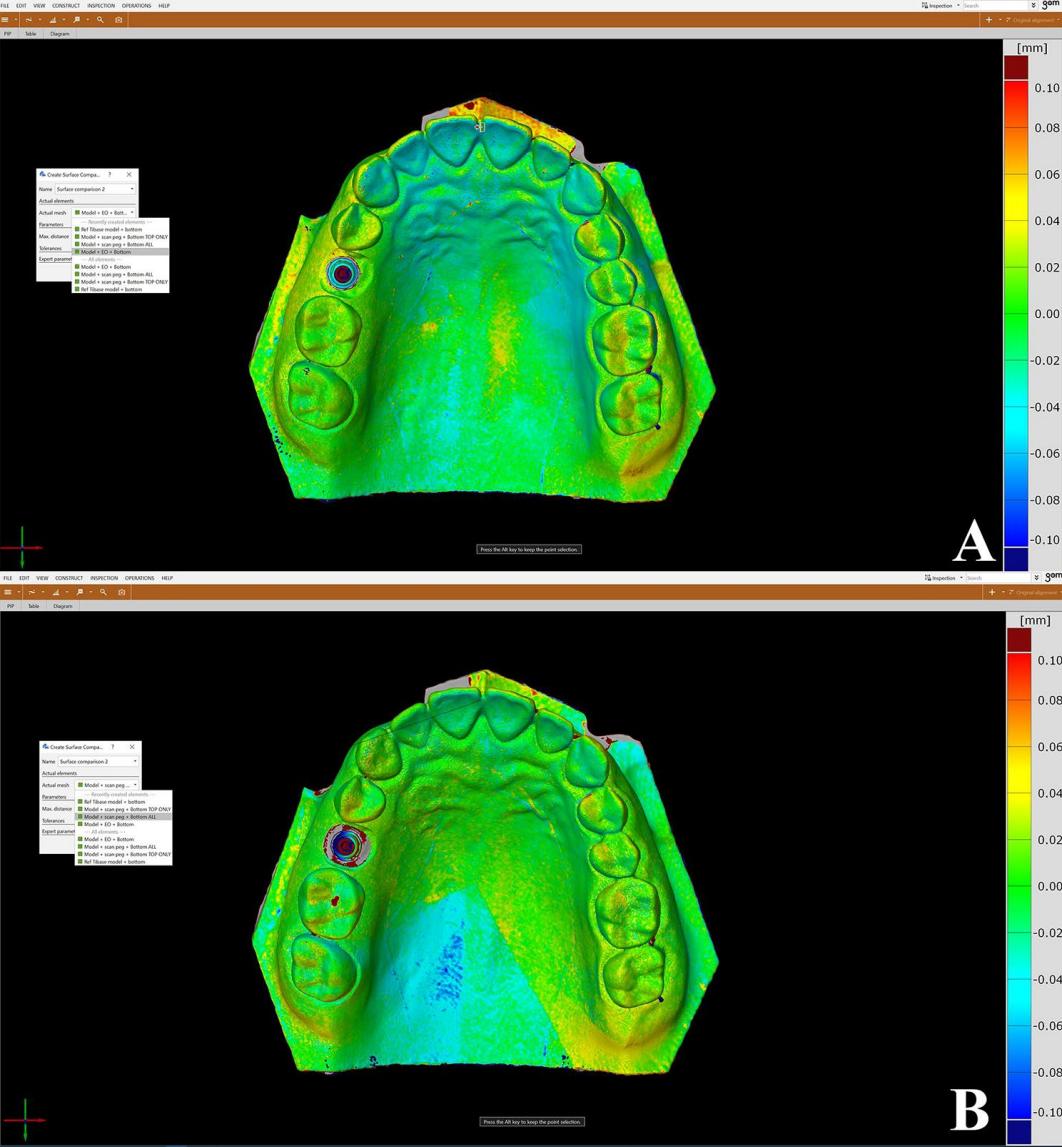
Local best-fit alignment of test and reference scans on 3D meterology software by selecting all teeth except TB site. The green color on the teeth showed zero deviation between the aligned test and reference scans, while the TB site showed red to blue color refering to the deviation that need to be analyzed (**A**) SB and reference scan, (**B**) HA-SP and reference scan

**Fig. 9:**
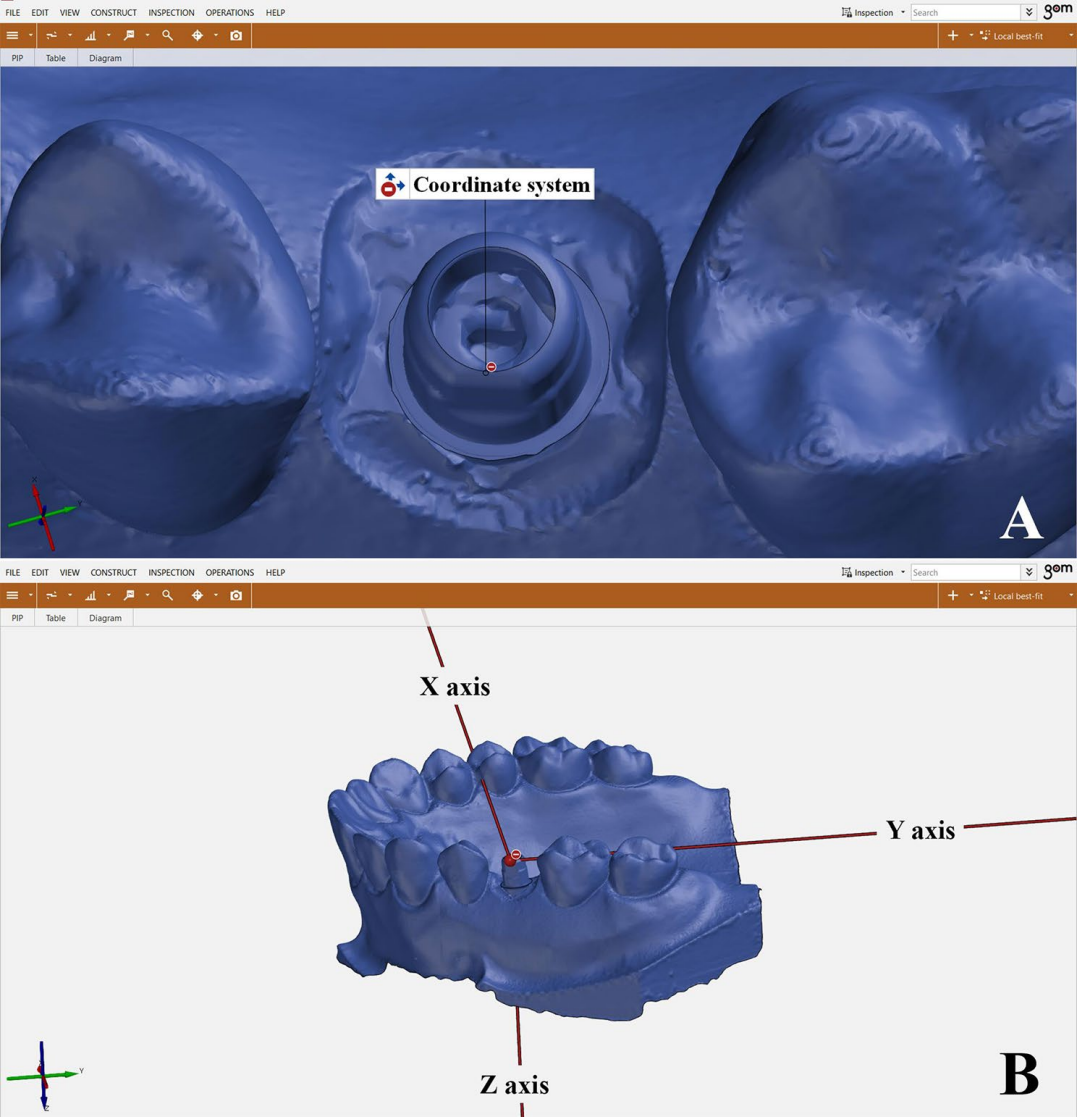
**A**. A coordinate system originating from a selected point on the occlusobuccal surface of the TB was used, **B.** XYZ axes resembled buccopalatal, mesiodistal and occlusocervical axes respectively

The software’s “pre-alignment” tool was used to initially align the received STLs. The “local best-fit” tool was then used to select all teeth other than the TB site for further alignment. (Fig. 8A, B) A coordinate system was constructed and applied during the whole analysis to calculate the angular and linear deviation of the TBs position. The XYZ axes were oriented on each scan originating from a selected point on the occlusobuccal surface of the TB so that the buccopalatal axis was the X axis, the mesiodistal axis was Y axis and the occlusocervical axis was Z axis. These coordinates were used to make sure that the TBs were always set to the same axes for subsequent comparisons. (Fig. 9A, B)

### Measurement of angular deviations

**Fig. 10:**
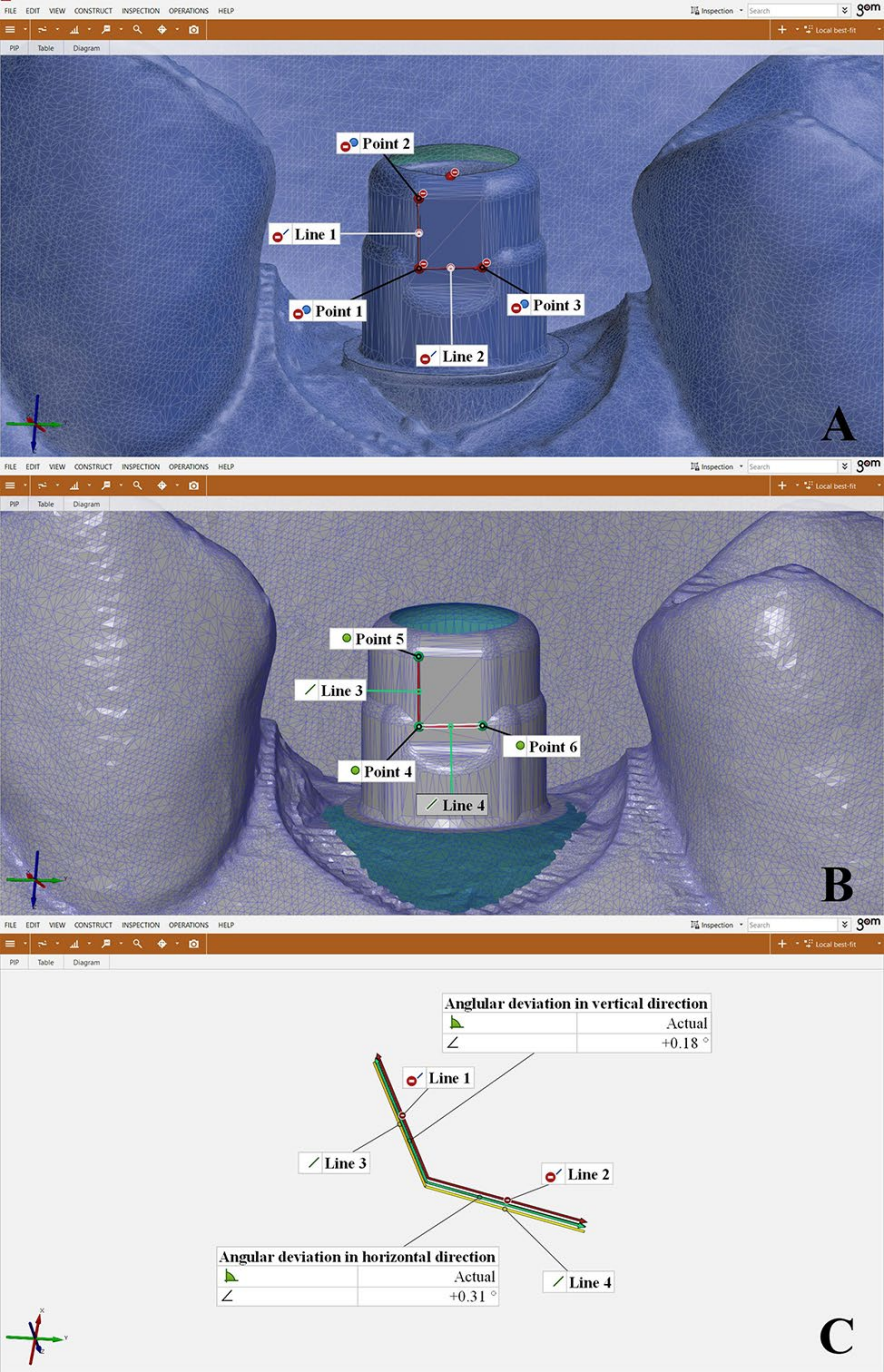
Angular deviation analysis **A**. Points and lines created on reference scan, **B**. Points and lines created on test scans, **C**. Angles between line 1 and line 3 resembled angular deviation in vertical direction and angles between line 2 and line 4 resembled angular deviation in horizontal direction

Angular deviations were measured in a vertical direction and a horizontal direction by measuring the angle between predetermined lines traced on the test TB compared to the reference TB. The surface of the scans was presented as series of flat polygons or triangles virtually to allow for appropriate point selection [2]. Points 1 and 2 were constructed to draw line 1 on occlusocervical direction of the reference TB and points 1 and 3 were constructed to draw line 2 on mesiodistal direction of the reference TB. (Fig. 10A) Points 4 and 5 were constructed to draw line 3 on occlusocervical direction of the test TB and points 4 and 6 were constructed to draw a line 4 on mesiodistal direction of the test TB. (Fig. 10B) Then, the angles between line 1 on reference scan and line 3 on the test scan were calculated resembling angular deviation in vertical direction. Also, the angles between line 2 on reference scan and line 4 on the test scan were calculated resembling angular deviation in horizontal direction. (Fig. 10C)

### Measurement of linear deviations in XYZ axes

**Fig. 11:**
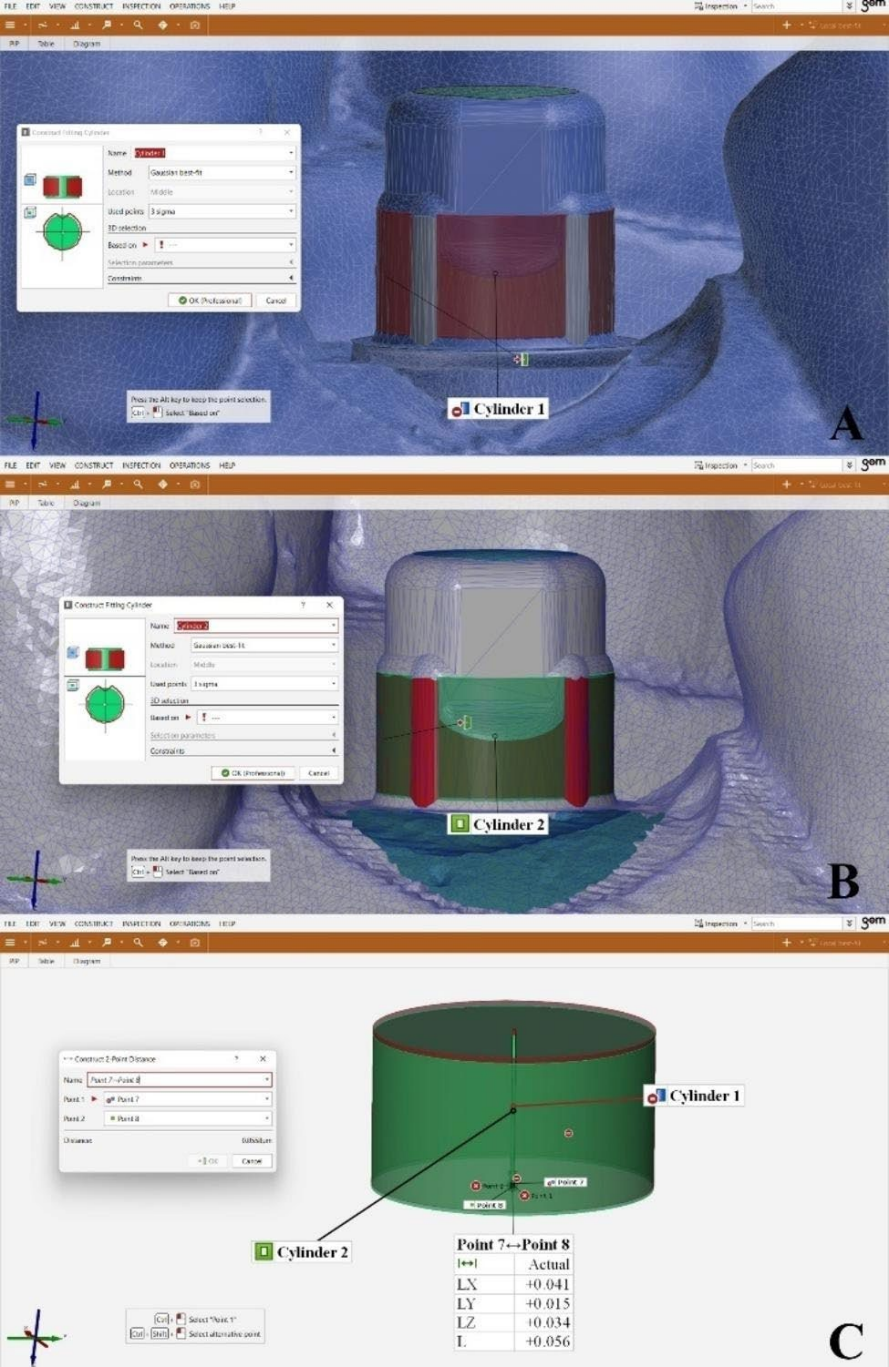
Linear deviation analysis (**A**) Fitting cylinder created on reference scan, (**B**) Fitting cylinder created on test scans, (**C**) A 2-point distance was constructed between both fitting cylinders’ center points corresponding to linear deviations in XYZ axes

Regarding the linear deviations, one fitting cylinder was constructed for each TB on the reference and the test scans. This fitting cylinder was constructed using the “Gaussian best-fit” method by selecting all cervical surface of the TB to guarantee repeatable cylinder’s center points and remove human-created mistakes affecting positional transfer accuracy measurements [28]. (Fig. 11A, B) A 2-point distance (point7-8) was constructed between both fitting cylinders’ center points corresponding to linear deviations in XYZ axes on the reference and the test scans to be compared. (Fig. 11C) The 3D linear deviation value was calculated according to the following formula: 3D linear deviation = $(\sqrt{{X}^{2}+{Y}^{2} +{Z}^{2}}$ ) [9, 17, 29, 30].

Low deviation value indicated a high degree of 3D matching of aligned files, which was translated to high trueness and precision.

### Statistical analysis

Statistical analysis was done by using a statistical software program (IBM SPSS Statistics, v25; IBM Corp). Kolmogorov-Smirnov test of normality showed no significant difference in the distribution of the variables; hence parametric statistics was used. Comparisons were done between two studied independent normally distributed variables using independent sample t test. When Levene’s test for equality of variances was significant, Welch’s t-test was applied. Statistical significance was tested at *P* value < 0.05.

## Results

Regarding the trueness, significant differences were found amongst the tested groups in vertical and horizontal angular deviation, linear deviation in each axis and 3D linear deviation in XYZ axes. SB group showed statistically significant less deviation values compared to HA-SP group (*P* < 0.001) as shown in Table 1. Thus, SB group had higher trueness values than HA-SP group.

**Table 1:** Comparison of trueness between SB group and HA-SP group

	Group		Test of significance	*P* value
	SB (n = 10)	HA-SP (n = 10)		
**Angular deviation in vertical direction (°)**				
- Min – Max - Mean ± SD - Standard error of mean - 95% CI of the mean - 25th Percentile − 75th Percentile	0.350–0.410 0.379 ± 0.023 0.007 0.363–0.395 0.360–0.400	1.590–1.780 1.676 ± 0.073 0.023 1.624–1.728 1.600–1.750	t_(W)(df=10.734)_ = 53.482	*P* < 0.001
**Angular deviation in horizontal direction (°)**				
- Min – Max - Mean ± SD - Standard error of mean - 95% CI of the mean 25th Percentile − 75th Percentile	0.220–0.430 0.288 ± 0.071 0.023 0.237–0.339 0.220–0.350	0.500–0.650 0.571 ± 0.044 0.014 0.539–0.603 0.540–0.600	t_(df=18)_ = 10.658	*P* < 0.001
**Linear deviation in X axis (μm)**				
- Min – Max - Mean ± SD - Standard error of mean - 95% CI of the mean - 25th Percentile − 75th Percentile	0.001–0.005 0.002 ± 0.001 0.000 0.001–0.003 0.001–0.003	0.153–0.167 0.157 ± 0.005 0.002 0.154–0.161 0.154–0.160	t_(W)(df=10.507)_ = 94.452	*P* < 0.001
**Linear deviation in Y axis (μm)**				
- Min – Max - Mean ± SD - Standard error of mean - 95% CI of the mean - 25th Percentile − 75th Percentile	0.001–0.005 0.002 ± 0.001 0.000 0.001–0.003 0.001–0.003	0.064–0.067 0.065 ± 0.001 0.000 0.064–0.066 0.064–0.066	t_(df=18)_ = 108.393	*P* < 0.001
**Linear deviation in Z axis (μm)**				
- Min – Max - Mean ± SD - Standard error of mean - 95% CI of the mean - 25th Percentile − 75th Percentile	0.053–0.055 0.054 ± 0.001 0.000 0.053–0.055 0.053–0.055	0.063–0.065 0.064 ± 0.001 0.000 0.063–0.065 0.063–0.065	t_(df=18)_ = 26.678	*P* < 0.001
**3D linear deviation (μm)**				
- Min – Max - Mean ± SD - Standard error of mean - 95% CI of the mean - 25th Percentile − 75th Percentile	0.053–0.055 0.054 ± 0.001 0.000 0.053–0.055 0.053–0.055	0.178–0.190 0.182 ± 0.004 0.001 0.179–0.185 0.179–0.184	t_(W)((df=9.742)_ = 89.841	*P* < 0.001

*Min – Max* Minimum – Maximum; *SD* Standard Deviation; *CI* Confidence interval; *t* Independent samples t test;

*W* Welch test; *df* degree of freedom

For the precision, significant differences were found amongst the tested groups in angular deviation in vertical direction. SB group showed statistically significant less angular deviation in vertical direction compared to HA-SP group *(P* < 0.001). However, no significant difference was found between the tested groups regarding the angular deviation in horizontal direction (*P* = 1.000). Moreover, significant differences were found amongst the tested groups in linear deviations in each axis as well as 3D linear deviation in XYZ axes. SB group showed statistically significant less linear deviation values in each axis and 3D linear deviation value compared to HA-SP group (*P* = 0.020, < 0.001, = 0.010, = 0.009 respectively) as shown in Table 2. Thus, SB group had higher precision values than HA-SP group except for the angular deviation in horizontal direction that was the same precision.

**Table 2:** Comparison of precision between SB group and HA-SP group

	Group		Test of significance	*P* value
	SB (n = 10)	HA-SP (n = 10)		
**Angular deviation in vertical direction (°)**				
- Min – Max - Mean ± SD - Standard error of mean - 95% CI of the mean - 25th Percentile − 75th Percentile	0.110–0.360 0.248 ± 0.087 0.028 0.186–0.310 0.180–0.300	0.400–0.860 0.641 ± 0.163 0.052 0.524–0.758 0.530–0.800	t_(df=18)_ = 6.711	*P* < 0.001
**Angular deviation in horizontal direction (°)**				
- Min – Max - Mean ± SD - Standard error of mean - 95% CI of the mean - 25th Percentile − 75th Percentile	0.290–0.790 0.447 ± 0.178 0.056 0.320–0.574 0.320–0.580	0.020–0.710 0.447 ± 0.247 0.078 0.270–0.624 0.270–0.690	t_(df=18)_ = 0.000	*P* = 1.000
**Linear deviation in X axis (μm)**				
- Min – Max - Mean ± SD - Standard error of mean - 95% CI of the mean - 25th Percentile − 75th Percentile	0.024–0.046 0.038 ± 0.007 0.002 0.032–0.043 0.037–0.042	0.024–0.101 0.065 ± 0.031 0.010 0.043–0.087 0.030–0.093	t_(W)(df=10.055)_ = 2.775	*P* = 0.020
**Linear deviation in Y axis (μm)**				
- Min – Max - Mean ± SD - Standard error of mean - 95% CI of the mean - 25th Percentile − 75th Percentile	0.000-0.018 0.010 ± 0.006 0.002 0.006–0.014 0.005–0.014	0.017–0.060 0.035 ± 0.015 0.005 0.024–0.046 0.021–0.049	t_(W)(df=11.566)_ = 4.813	*P* < 0.001
**Linear deviation in Z axis (μm)**				
- Min – Max - Mean ± SD - Standard error of mean - 95% CI of the mean - 25th Percentile − 75th Percentile	0.022–0.042 0.032 ± 0.006 0.002 0.028–0.036 0.029–0.034	0.019–0.102 0.063 ± 0.031 0.010 0.041–0.085 0.032–0.091	t_(W)(df=9.588)_ = 3.196	*P* = 0.010
**3D linear deviation (μm)**				
- Min – Max - Mean ± SD - Standard error of mean - 95% CI of the mean - 25th Percentile − 75th Percentile	0.033–0.061 0.051 ± 0.008 0.002 0.045–0.056 0.049–0.055	0.037–0.154 0.098 ± 0.045 0.014 0.066–0.130 0.047–0.133	t_(W)(df=9.588)_ = 3.278	*P* = 0.009

*Min – Max* Minimum – Maximum; *SD* Standard Deviation; *CI* Confidence interval; *t* Independent samples t test;

*W* Welch test; *df* degree of freedom

## Discussion

The positional transfer accuracy (trueness and precision) of TB abutment provided by one-piece SB was different from that provided by two-piece HA-SP in terms of angular and linear deviations of TB position and accordingly the null hypothesis was rejected. The current study analyzed the positional transfer accuracy of TB provided by two scan body designs only as these were the only designs compatible to the used Neoss implant.

A laboratory scanner with 2.1 μm accuracy according to DIN EN ISO 12836.2015 was employed in the current study to standardize the digitizing process by reducing human errors that could occur by intraoral scanners, since a laboratory scanner automatically rotates the scanned object into various locations to provide the ideal illumination for the best data acquisition [31]. This scanner utilized to create the reference and test STL files has also been recommended in previous studies on deviation analyses [24, 25]. All the scans were made on the same day of printing the model to overcome the dimensional changes that may occur to the printed model over time, thus obtaining accurate superimposition of the scans [26].

Regarding the accuracy analysis, trueness and precision were analyzed based on ISO 5725-1 [22]. The ISO technique was chosen as the standard methodology, which the authors of the current study believe will be useful for further comparison of these findings with other studies [32].

The used 3D metrology software to make superimpositions of different scans had been widely used for evaluation of accuracy [15–17, 28]. This software program was classified as class1 (lowest measurement deviations) by the PTB and the NIST [33]. Although the best-fit alignment is often employed for analyses of accuracy, it has little shortcomings; the software looks for the perfect superimposition of two surface scans with the minimal deviation between all surface points [16]. The distance between two corresponding points may as a result be underestimated [34]. Thus, the current study utilized a local rather than an overall best-fit alignment except for the site of TB. Following the local best-fit algorithm, selected points on the TBs were used for the comparisons to reduce the underestimate [16].

Previous studies [15, 16] investigated the intraoral scan accuracy of HA-SP by measuring distance and angular deviations. However, these studies did not investigate which SB was more accurate in transfer of 3D implant position. Thus, it was evaluated in the current study.

The present study measured the angular deviation of TBs position using standardized points of measurement that were selected on occlusocervical and mesiodistal directions of TBs to reduce the possibility of random measurement errors in 3D analysis. These points were selected after virtual presentation of the scan surface as series of flat polygons or triangles as mentioned in a systematic review by Mizumoto and Yilmaz [2]. Additionally, the study measured the linear deviation values of TBs in XYZ axes by forming fitting cylinders on the cervical area of TBs similar to a previous study by Atalay et al. [17] who created fitting circles 3 mm below and parallel to the upper plane of HA-SP to evaluate the impact of implant location and operator on the scan accuracy provided by HA-SP. This technique may be advantageous as all available information can be used for comparisons and it did not depend on certain points that may not be visible as reported by Atalay et al. [17].

In the current study, SB group showed lower 3D deviation value than HA-SP group in terms of both trueness and precision. However, Yilmaz et al., [16] performed an in vitro study on intraoral scan accuracy using SB and HA-SP using points selected on certain landmarks and reported that SB had lower linear deviation than HA-SP in terms of trueness in one point only which was middle point on buccal coronal slope. Additionally, no significant difference between SB and HA-SP were reported in their study in terms of precision. This difference in the findings may be attributed to dissimilarities in the methodology between the studies.

The positional transfer accuracy of TB abutment provided by one-piece SB was higher than that provided by two-piece HA-SP and this may be related to several factors. The variability in the design characteristics between the 2 mentioned scan bodies may have led to the difference in accuracy. HA-SP was formed of 2 pieces and the seating of the 2 pieces may be influenced by the manufacturing tolerances, that could have an effect on the accuracy as reported in previous in vitro studies [6, 35]. Furthermore, Donmez et al., [18] reported that the absence of a flat surface in HA-SP might have caused the higher deviation values upon investigation of the congruence between the meshes of HA-SP obtained by four different intraoral scanners used in their study and the corresponding library file. This may explain the higher deviation values of HA-SP in comparison to SB in the present study. Additionally, the difference in the implant connection’s material of each scan body was another contributing factor affecting the positional transfer accuracy of implant as reported by Schmidt et al., [5] that may contribute to the final prosthetic misfit. The one-piece SB was made of PEEK and its implant connection was in titanium. However, the HA was made entirely of PEEK, its implant connection was in PEEK and its SP was made of medical grade acrylic-based polymer. Furthermore, the difference in the installation method of each scan body was another factor influencing the transfer accuracy as one-piece SB was tightened directly on implants, but the SP in the HA-SP system was fastened to HA through autorotational frictional means without screw [15–18].

### Limitations and recommendations

The limitations of this in vitro study included that the model utilized in the current study did not accurately represent the intraoral circumstances; teeth, soft tissues, and the existence of saliva or blood [27, 36]. The use of a laboratory scanner is another limitation, thus future studies should be conducted utilizing an industrial high-accuracy scanner [37]. The used CAD software “best-fit matching” tool in the current study for generating the reference and test scans is considered as a limitation due to the lack of data in the literature about its margin of error, therefore further use of other CAD software programs should be done. In addition, the reference scan was produced by aligning a TB from the CAD library to the original scanned model and this may also introduce some degree of error in the reference scan. Furthermore, angular and linear deviations may influence the proximal and occlusal contacts of definitive implant-supported restorations [15, 16]. Therefore, clinical performance and positional accuracy of definitive crowns regarding proximal contacts with the neighboring teeth and occlusal contacts with the opposing teeth should be further evaluated using both scan bodies through clinical studies as well as the effect of HA-SP on the contouring of the soft tissues.

## Conclusions

Within the limitations of this in vitro study, the following conclusions were drawn:

1. One-piece SB showed less angular and linear deviation in the 3D transfer of TB position than two-piece HA-SP indicating a higher degree of accuracy of SB.
2. Different designs, materials and installation methods of the scan bodies could affect the 3D positional transfer accuracy of TB.

## Data Availability

The datasets used and/or analysed during the current study are available from the corresponding author on reasonable request.

## List of abbreviations

3D: Three-dimensional
HA-SP: Healing abutment-scan peg
NIST: National Institute of Standards and Technology
PEEK: PolyEtherEtherKetone
PTB: Physikalisch-Technische Bundesanstalt
SB: Scan body
STL: Standard Tessellation Language
TB: Titanium base
